# Philosophy of Science Can Prevent Manslaughter

**DOI:** 10.1007/s11673-022-10198-4

**Published:** 2022-06-24

**Authors:** Andreas De Block, Pierre Delaere, Kristien Hens

**Affiliations:** 1 Centre for Logic and Philosophy of Science, Kardinaal Mercierplein 2 - box 3200, 3000 Leuven, Belgium; 2 Department of Microbiology, Immunology and Transplantation, Herestraat 49, 3000 Leuven, Belgium; 3 Department of Philosophy, Rodestraat 14, 2000 Antwerpen, Belgium

**Keywords:** Research ethics, trachea, Macchiarini, philosophy of science, ethics committees

## Abstract

In September 2020, the surgeon Paulo Macchiarini, who used stem cell technology to enable the transplants of artificial and donor trachea, was charged with aggravated assault in Sweden. In this comment, we argue that the Ethics Council of the Karolinska Institute should have considered issues from philosophy of science when they were brought to their attention, rather than dismiss them as irrelevant to research ethics. We demonstrate how conceptual issues of a philosophy-of-science-kind about clinical research and medical practice should be integral to research ethics.

## Background

The infamous case of Paolo Macchiarini and his use of stem cell technology to enable the transplants of artificial and donor trachea has been well documented in several popular books, documentaries, and academic publications. It has become clear that Macchiarini embellished research data, performed deadly experiments on patients, and bullied his more critical colleagues into silence (C. Elliott 2017; Hyvönen 2020). The breaches of research ethics at different stages are mind-blowing. It is fascinating how Macchiarini, who was called “Dr Con Man” in a Guardian article outlining the case (Rasko and Power 2017), could trick academic publishers, ethics committees, and departments into believing that he could cure patients needing a new trachea.

Nevertheless, the emphasis on the fraudulent behaviour of the charismatic Macchiarini risks obscuring more fundamental questions about the relationship between biomedical science, philosophy, and ethics. Here, we want to focus on two such questions. What is the relationship between philosophy of science and biomedical ethics? How is this relationship relevant for the assessment of experimental practices by research ethics councils? The authors of this paper were inspired by a response that one of us received when, in 2015, he notified the Ethics Council of the Karolinska Institute of the possibility of fabricated data and fraudulent behaviour by Macchiarini, who was at that time employed at the Karolinska Institute (KI). The conclusion to the response goes as follows “We find that the issues raised by Professor Delaere are of a philosophy-of-science kind rather than of a research-ethical kind. Accordingly, the Ethics Council concludes that Professor Delaere’s allegations of scientific misconduct are unfounded.”^1^ This phrase suggests that philosophy of science and research ethics have to be seen as two different disciplines with little, if any, overlap. Our paper will argue that this view is incorrect and—as the Macchiarini case indicates—dangerous. The integrity of medical research and medical interventions often requires conceptually sound proposals and a profound understanding of the dynamics of science. Hence, arguments that build upon philosophy of science can be, and often are, morally relevant.

In what follows, we first sketch the broad lines of the Macchiarini scandal. Then we analyse and evaluate the Ethics Council’s dismissal of Delaere’s arguments. One interpretation of the Ethical Council’s response is that by qualifying these arguments as *philosophy of science*, the Ethical Council can dismiss them *with the help of philosophy of science*. We will argue that this makes sense but that the strength of the dismissal depends on the strength of the counterargument. However, in this particular case, the counterargument was of questionable quality. A second interpretation is *that philosophy of science is irrelevant for research ethics and medical ethics*. Such ground for dismissal is flawed, we argue, partly because the subdivision of disciplines such as philosophy of science, research ethics, and medical ethics is arbitrary and the result of historical contingencies. Additionally, we will counter the claim that, as many significant innovations may seem at odds with current knowledge, prohibiting experimental research or protocols based on conceptual issues or feasibility concerns hampers progress in biomedical science. To conclude, we advocate a further rapprochement of philosophy of science and research ethics, both concerning collaboration between academics working in the fields and concerning the education of philosophers, medical ethicists, medical doctors, and specialists in ethics committees.

## The Macchiarini Scandal

In 2008, head and neck surgeon Paolo Macchiarini started treating individuals who needed replacement of a diseased windpipe segment. His treatment was unique because it used an artificial organ engineered with the patients’ stem cells. By 2015, he had treated around twenty patients with this seemingly revolutionary therapy. In medical literature and the popular media, this achievement was heralded as the first clinically successful use of an artificially engineered organ and the beginning of a new era of stem cell–engineered organ regeneration.

From early on, several researchers remained unconvinced. In 2009, Pierre Delaere, a thoracic surgeon himself, voiced concerns in *The Lancet* over the results that Macchiarini and colleagues had published a few months earlier in that same outlet (Delaere and Hermans 2009). According to Delaere, the stem cell–based engineering of a functional artificial windpipe was impossible, given the state-of-the-art knowledge. A first issue was that, according to Macchiarini et al., the cells of a donor trachea had been replaced by recipient cells after stem cells of the recipient had been applied to a cadaver donor trachea. Delaere pointed out that this amount of regeneration in the absence of any blood supply had never been observed before in experimental or clinical conditions.

Moreover, Macchiarini reported that the “regenerated” trachea could be used for airway repair without restoring the blood supply. Delaere considered this very improbable. After all, organs and composite tissues are transplantable (or can be used for reconstruction) because the perfusing blood vessels can be preserved or repaired. The trachea, however, is one of the few organs that is generally seen as (currently) untransplantable because its complex blood supply cannot be directly repaired with microvascular techniques.

Delaere’s concerns were published as a comment in *The Lancet* but with minimal impact on Macchiarini’s career. He continued doing trachea transplants and (co-) authoring high profile publications on the technique he claimed to have developed. As a matter of fact, in 2010, less than a year after Delaere’s comment was published, Macchiarini was recruited as a visiting professor at Karolinska Institute (KI), where he continued his clinical work at the university hospital. Only in 2016, after numerous complaints and procedures, did KI decide not to renew Macchiarini’s contract. In the meantime, many of Macchiarini’s patients had died an appalling death. For that reason, Swedish prosecutors decided in September 2020 to sue Paulo Macchiarini for aggravated manslaughter.

Leading medical centres and institutions created an environment where Macchiarini’s misconduct could occur. *The Lancet* published papers by Macchiarini and co-authors on their “successes” with tracheal transplants in 2011, 2012, 2013, and 2014, despite the warnings of other specialists. These papers were published even though the *New England Journal of Medicine* had rejected at least two of them, in part because they lacked any visualization of the “regenerated” trachea and were therefore considered to be not credible enough to warrant publication. In March 2012, *The Lancet*’s deputy editor David Holmes even wrote an editorial entitled “Paolo Macchiarini: Crossing frontiers,” in which Macchiarini’s successes and the revolutionary potential of Macchiarini’s work were emphasized (Holmes 2012). At the time of writing of this paper, this editorial is still available online without update. Interestingly, *The Lancet* stopped publishing submitted letters or comments critical of Macchiarini’s work after 2009, rejecting many after in-house review. Only in 2016 did *The Lancet* publish an expression of concern about Macchiarini’s 2011 paper (*The Lancet* editors 2016), and in 2018 they retracted the 2011 paper and a paper from 2012 (*The Lancet* 2018). We note that this is not the first time *The Lancet* published fringe and fraudulent research. It provided a highly reputed home for Andrew Wakefield’s claims regarding the link between autism and MMR vaccines, which fueled vaccine hesitancy and may thus have contributed to a rise in illnesses that would have been preventable. The study was only retracted after twelve years (Eggertson 2010). Nor was it the last time. *The Lancet* published and then retracted a study on the use of hydroxychloroquine to treat COVID-19. Academic publishers may be forgiven for making mistakes. Mistakes and failures are part and parcel of scientific practice. What is less forgivable is not learning from them, especially when they have led to deaths and injuries. A humble excuse is the least one would expect. KI was no less instrumental in Macchiarini’s misconduct. Not only did it offer a professorship to Macchiarini in 2010 (*after* serious concerns had been voiced), the KI also defended Macchiarini against internal whistleblowers who had assisted with the transplants or were involved with the aftercare of Macchiarini’s patients. After the whistleblower’s accusation that Macchiarini had misrepresented the success of the operations, omitting or even fabricating data in his published articles, KI asked the chief medical officer of Karolinska University Hospital to report the whistleblowers to Swedish police. KI claimed that the whistleblowers had violated the law and the patients’ privacy when they went through the patients’ charts and submitted their appeals for an investigation to KI and the Central Ethical Review Board.

From the very beginning, KI’s Ethical Council did not seem eager to investigate, let alone condemn Macchiarini. However, their handling of an allegation by Delaere deserves further analysis.

## Is Philosophy of Science Irrelevant for Research Ethics?

In April 2015, Pierre Delaere wrote to the Ethics Council of Karolinska Institute to express his concerns about Macchiarini. He focused on his severe doubts about the very possibility of stem cell–based regeneration of the trachea. The Ethics Council responded dismissively to these concerns. They concluded their response, as cited above, as follows:

We find that the issues raised by Professor Delaere are of a philosophy-of-science kind rather than of a research-ethical kind. Accordingly, the Ethics Council concludes that, on the backdrop of the examined issues, Professor Delaere’s allegations of scientific misconduct are unfounded.

As one reviewer noticed, this response is maybe an easy excuse for not further investigating Delaere’s major concerns. Yet, we will consider it here as a genuine worry, because it raises interesting questions concerning the relationship between medical research ethics and philosophy of science.

In the point-by-point rebuttal in the Ethics Council’s response, the Ethics Council itself used arguments and insights from philosophy of science to refute Delaere’s allegations. We give two examples. First and in response to Delaere’s claim that Macchiarini and some of his co-workers fabricated data, the Ethics Council makes the Kuhnian point that observations are often theory-laden and that mitigating this problem by using blinded procedures is very difficult with surgical interventions in just a few patients. Secondly, the Ethics Council repudiates Delaere’s claim that Macchiarini’s “findings” are at odds with medical science. They underscored, like true Popperians, that “empirical findings might contradict current theories—otherwise we would not be able to make progress in, e.g. the medical sciences.”

This response requires further unpacking. First, the Ethics Council seems to think that since Delaere’s concerns are of a “philosophy of science kind,” they can be best addressed and refuted with the help of philosophy of science. To some extent, this makes sense. The best arguments against views in a particular discipline can usually be found within that discipline. Although physicists may develop interesting critiques of multiple sclerosis research, we correctly assume that the best critiques against such research can be found in fields directly related to biomedical theory (see below for a couple of important caveats). Likewise, the philosophy of science literature contains many papers and monographs that directly argue against particular positions in philosophy of science. That being said, this does not entail that the actual response of KI’s Ethics Council is convincing. After all, although the conceptual and feasibility issues that Delaere raised touch upon core topics in philosophy of science, and even though KI’s research council may have correctly identified these core topics, the refutations are argumentatively dubious.

Let us consider the two responses that we already mentioned. First, one could indeed argue that observations are often theory-laden, as the Ethics Council claims. Although there is some debate on whether most perception is influenced by theoretical beliefs (Brewer and Lambert 2001), few philosophers would deny that attention and the interpretation of observations are partially steered by all sorts of beliefs that the observer holds. For example, to understand neuroimaging data as providing information about the cognitive processes, one needs to have a set of theoretical beliefs on how imaging technology and the nervous system work (Aktunc 2021). Much less obvious is the idea that the theory-ladenness of observations substantially blurs the line between fabricated data and correctly obtained observational data. If scientists are presented with visualizations and still disagree on what they observe, this might be blamed on the theory-ladenness of observation. However, this is not what happened in this case. What did happen was that one scientist, Macchiarini, claimed to have observed something—something which another scientist, Delaere, said was impossible, considering what is known about the human body.

Moreover, observations and interpretations of observations that are steered or coloured by novel, revolutionary theories are, all else being equal, more suspect than observations that are laden with the assumptions of scientific theories with a long and robust track record of epistemic successes. After all, we can assume that the latter tell us (approximately) how the human body works. In that light, the response of KI’s Ethics Council contrasts unfavourably with the decision of the *New England Journal of Medicine* to reject Macchiarini’s papers because they did not include the necessary visualizations of the allegedly regenerated trachea.

The second response builds on Karl Popper’s idea that science progresses through falsifying theories and hypotheses that were previously thought to be correct. Now Delaere did not deny that this is how science proceeds. He did bring to the fore that the shared data were blatantly insufficient to falsify any medical theory, simply because very few of the raw data were shared with the scientific community. Moreover, one of Popper’s main points is that science should not immunize itself against its falsification. Therefore, it is striking that the Ethics Council seems to legitimize an immunization tactic deployed by Macchiarini. No regenerated tissue was found in autopsied or re-operated patients. Moreover, the Council accepted Macchiarini’s defence that other interventions probably destroyed it in one case. In a second case, such regenerated tissue was allegedly observed by a local pathologist. The Ethical Council simply notes that “it has no reason to doubt that the local pathologist found epithelisation and neovasculation,” although it seemingly did not have strong evidence that the local pathologist found anything. Admittedly, our assessment of the Ethics Council’s judgement is informed by the later developments in this scandal. Nonetheless, if the responses are meant to address the expressed concerns with the tools and ideas of philosophy of science, they fall short of being fully convincing.

More importantly, the Ethics Council’s response is based upon an assumption that if philosophy of science is instrumental in settling (or maybe even just exploring) an issue, the issue must be “of a philosophy-of-science kind” and not primarily “of a research-ethical kind.” The four-page letter of the Research Council concludes by stating that the allegations raised against Macchiarini are unfounded *because* they are not of a research-ethical kind. Again, there is something to be said for the view that the allegations involve issues that are regularly associated with philosophy of science. For instance, Delaere’s concerns with how the interventions of Macchiarini and his team fit within extant theories could be mapped onto debates in philosophy of science over revolutionary science. Nevertheless, we think that the Ethics Council wrongly concludes from this that the allegations are not of a research-ethical kind. We first explain why this conclusion is wrong, then why it is not an innocent mistake.

Boundaries between fields and subfields, or disciplines and subdisciplines, are at least to some extent historical contingencies that could have been different. The way our academic disciplines are currently defined and organized is somewhat arbitrary (Østreng 2010). More often than not, fields have fuzzy boundaries and display overlaps with other fields. For example, history, sociology, and philosophy of science all deal with scientific revolutions and the role observations play in constructing theories (Hoyningen-Huene 1992). Furthermore, the fact that an issue can be approached rather successfully with the help of one discipline or field does not automatically imply that another field of discipline cannot assist in solving or resolving that issue. For instance, it would be wrong to argue that since pneumologists can understand a respiratory problem, oncological knowledge is irrelevant for understanding or treating the problem. Very often, so-called *interdisciplines* such as pneumo-oncology arise precisely because it is acknowledged that two or more disciplines can further our knowledge of a set of phenomena by creating synergies between their methods and insights (Leydesdorff 2021). Likewise, acknowledging the value-ladenness of science and scientific research has given rise to the “science and values” field. This field brings together methods and insights from, among others, philosophy of science and ethics. Particularly relevant in this case is that this field emphasizes how research choice often should be morally evaluated. For instance, extensive literature exists on how the weighting of evidence should also involve so-called non-epistemic values that pertain to the harm that is involved in incorrectly accepting (false positive) or incorrectly accepting (false negative) a hypothesis (Douglas 2000; Elliott and Richards 2017). Hence, Delaere’s expert assessment that Macchiarini’s research was unlikely to succeed has important implications for research and medical ethics, given that acting on a false positive would probably have life-threatening implications. Revolutionary science is associated with much more uncertainty than “normal science.” This fact should inform the cost-benefit analysis of technologies that result from such revolutionary research (Mayo and Hollander 1994). Even just exploring the possibility of a medical intervention that is at odds with our current and foreseeable future knowledge risks wasting research resources, a waste that is often ethically problematic. Opportunity costs are sometimes also moral costs. Ethics Committees evaluating research protocols employing statistical methods pay close attention to sample sizes calculation because getting this wrong would risk wasting participants’ time with futile research endeavours. Surely we can expect that they should also assess the conceptual soundness of experimental methods and be open to investigating complaints about such soundness. After all, what is at stake here is more than a risk of wasting people’s time: most of Macchiarini’s trachea transplant patients died, and many of them were in agonizing pain.

In other words, even though the issues raised by Delaere may have been “of a philosophy-of-science kind,” they still point to important research-ethical issues. They do so not just because philosophy of science also deals with non-epistemic values but also because research ethics focuses on the interplay and intertwinement of epistemic and non-epistemic values. Honesty and the Mertonian values of disinterestedness and communality are both morally and epistemically desirable. This is no coincidence, of course, since research ethics is concerned with scientific research, and society values science in no small part because of the epistemic goods it delivers.

## Scientific Progress Versus Overzealous Ethics Committees

In the “regenerated” trachea case, the *New England Journal of Medicine* had already rejected papers by Macchiarini, based on the lack of mechanistic evidence provided by Macchiarini and his co-authors. It is strange and even morally questionable that this lack of mechanistic evidence was dismissed as ethically irrelevant by KI’s Ethics Council (Andersen 2012). Suppose mechanistic evidence is unavailable or its absence cannot be sufficiently explained. In that case, this should raise ethical red flags: it can suggest that the research or the experimental method is futile or dangerous, or even the outcome of fraudulent intent. In other words, strict ethical scrutiny of improbable research and improbable research results is commendable. How can one explain the reluctance of KI’s Ethics Council? We can speculate that this had to do with Macchiarini’s reputation. Another possibility is that the Ethics Council’s leniency was motivated by the fact that KI had just received a donation of US$50 million from the Hong Kong–based businessman Ming Wai Lau to establish a research centre in regenerative medicine. However, a more charitable reading is also possible. The Council may have genuinely feared that such scrutiny would stop or hamper medical progress. This can be a legitimate concern because the past has shown that novel techniques (including surgical interventions) turn out to be beneficial even when there remain significant gaps in our knowledge of how they bring the benefits about. For instance, it is still unknown why airplanes stay in the air, but this aerodynamic lift enigma has not stopped scientists and engineers from designing and developing them. In the context of the Macchiarini case, the example of heart transplantation has often been mentioned. Indeed, the first heart transplants were unsuccessful and led to the death of those receiving them. If ethics committees had forbidden these transplants to proceed, the life-saving benefits that heart transplants have today would have been unavailable.

That being said, we believe that a philosophy of science approach can help demarcate procedures like heart transplants from those like artificial tracheas with stem cells, or “head transplants” for that matter. Such demarcation is, in our view, part and parcel of the task of an ethics committee and the field of research ethics. They should not shy away from determining the allocation and the strength of the burden of proof, through an assessment of the health risks and benefits and other non-epistemic values (such as financial costs) (Todt and Luján 2017). Obviously, this also involves carefully weighing the evidence and counter-evidence for what is or seems at odds with existing knowledge. If the members of the ethics committees lack the necessary expertise to assess the available evidence or the proposed explanation and mechanisms, they should commission an investigation by qualified experts. Furthermore, during the last decade, philosophers of science have provided conceptual and semantic analyses that benefited stem cell research and oncology (Laplane et al. 2019). Such analyses can also be instrumental in assessing other revolutionary techniques or research protocols.

## Conclusion

Medical and research ethics, and philosophy of science, are often worlds apart, sometimes even geographically. Philosophers of science tend to be in philosophy departments, whereas research ethics specialists or bioethicists are often employed at medical departments. What the Macchiarini case shows is that such schism has potentially deleterious consequences.

The Macchiarini case, pernicious as it is, has taught the research community many vital lessons about research conduct. We have argued that one of its lessons should be to stop considering philosophical and research-ethical questions as belonging to categorically different domains. Research integrity can definitely be at stake if the science itself is conceptually unsound or even flaky. Conceptual and theoretical considerations have a direct impact on bioethical considerations. We think a cross-pollination of these disciplines will significantly aid scientific and biomedical practice. Such cross-pollination needs to happen in different ways. We hope that biomedical ethicists and philosophers of science can work together more closely across disciplinary and departmental boundaries. Training in biomedical ethics must contain a substantial portion of philosophy of science and vice versa. Biomedical ethics and research ethics courses for doctors and researchers in biomedical ethics should also include conceptual issues and other topics from philosophy of science and teach how these can enrich ethical considerations. Finally, ethics councils must not restrict themselves to overseeing research integrity regarding whether the appropriate ethical procedures are in place. They should also assess—or at least be able to assess—the soundness of the theoretical assumptions and constructs on which research and clinical experiments are based. This needs to be the case even when researchers or clinicians are considered stellar or when reputable organizations have already funded the projects.

## Footnotes

1 The entire response can be read here: http://retractionwatch.com/wp-content/uploads/2015/04/2445_001.pdf
